# Necroptosis Contributes to LPS-Induced Activation of the Hypothalamic-Pituitary-Adrenal Axis in a Piglet Model

**DOI:** 10.3390/ijms231911218

**Published:** 2022-09-23

**Authors:** Bei Zhou, Qilong Xu, Junjie Guo, Qinliang Chen, Qingqing Lv, Kan Xiao, Huiling Zhu, Jiangchao Zhao, Yulan Liu

**Affiliations:** 1 Hubei Key Laboratory of Animal Nutrition and Feed Science, Hubei Collaborative Innovation Center for Animal Nutrition and Feed Safety, Wuhan Polytechnic University, Wuhan 430023, China; 2 Department of Animal Science, Division of Agriculture, University of Arkansas, Fayetteville, AR 72701, USA

**Keywords:** necroptosis, hypothalamic-pituitary-adrenal axis, lipopolysaccharide, necrostatin-1, pigs

## Abstract

Stressors cause activation of the hypothalamic-pituitary-adrenal (HPA) axis and a systemic inflammatory response. As a newly proposed cell death manner in recent years, necroptosis occurs in a variety of tissue damage and inflammation. However, the role of necroptosis in HPA axis activation remains to be elucidated. The aim of this study was to investigate the occurrence of necroptosis and its role in HPA activation in a porcine stress model induced by *Escherichia coli* lipopolysaccharide (LPS). Several typical stress behaviors like fever, anorexia, shivering and vomiting were observed in piglets after LPS injection. HPA axis was activated as shown by increased plasma cortisol concentration and mRNA expression of pituitary corticotropin-releasing hormone receptor 1 (*CRHR1*) and adrenal steroidogenic acute regulatory protein (*StAR*). The mRNA expression of tumor necrosis factor α (*TNF-α*), interleukin-1β (*IL-1β*) and *IL-6* in the hypothalamus, pituitary gland and adrenal gland was elevated by LPS, accompanied by the activation of necroptosis indicated by higher mRNA expression of necroptosis signals including receptor-interacting protein kinase (RIP) 1, RIP3, and phosphorylated mixed-lineage kinase domain-like protein (MLKL). Furthermore, necrostatin-1 (Nec-1), an inhibitor of necroptosis, inhibited necroptosis indicated by decreased mRNA levels of *RIP1*, *RIP3*, *MLKL*, and phosphoglycerate mutase family member 5 (*PGAM5*) in the hypothalamus, pituitary gland and adrenal gland. Nec-1 also decreased the mRNA expression of *TNF-α* and *IL-β* and inhibited the activation of the HPA axis indicated by lower plasma cortisol concentration and mRNA expression of adrenal type 2 melanocortin receptor (*MC2R*) and *StAR*. These findings suggest that necroptosis is present and contributes to HPA axis activation induced by LPS. These findings provide a potential possibility for necroptosis as an intervention target for alleviating HPA axis activation and stress responses.

## 1. Introduction

Sepsis is a systemic inflammatory response syndrome that occurs during infection and is considered to be the leading cause of death in clinical patients [1]. A pathogenic or lipopolysaccharide (LPS) challenge usually leads to the production of a variety of pro-inflammatory cytokines, such as tumor necrosis factor α (TNF-α), interleukin-1β (IL-1β), and IL-6 [2]. These inflammatory cytokines can cross the blood–brain barrier or directly act on the neuroendocrine system, leading to the activation of the hypothalamic-pituitary-adrenal (HPA) axis, resulting in endocrine and metabolic disorders, thus aggravating stress injury [3]. The neuroendocrine and immune system form a complex and delicate neuroendocrine-immune network through the secretion of active substances, which jointly participate in maintaining the homeostasis of the internal environment [3]. In particular, the HPA axis plays an irreplaceable role in stress or infection [4]. Therefore, exploring the mechanism of HPA axis activation under stress is helpful to propose prevention and treatment strategies for inhibiting HPA axis activation and subsequent stress injury.

Neuronal cell death plays a crucial role in the development and homeostasis of the nervous system [5]. There is extensive evidence suggesting that dysregulation of cell death and progressive neuroinflammation may occur in infectious, traumatic, and ischemic brain injury, and various neurodegenerative diseases such as Alzheimer’s diseases (AD), Parkinson’s diseases (PD) and Huntington’s diseases [6,7]. The classical modes of cell death discussed in neurological diseases include apoptosis, necrosis, autophagy, and excitotoxicity [6]. Excitingly, necroptosis, a novel form of cell death proposed in recent years, has been shown to occur in various tissue injuries including the nervous system [8,9,10]. Necroptosis can be triggered by death receptors like tumor necrosis factor receptor 1 (TNFR1) [11]. TNFR1 activation leads to the recruitment and deubiquitylation of the receptor interacting protein kinase 1 (RIP1). The activated RIP1 then autophosphorates and recruits receptor interacting protein kinase 3 (RIP3) by interacting with the homologous domain, resulting in the phosphorylation of mixed lineage kinase domain-like protein (MLKL) and the formation of RIPK1/RIPK3/MLKL complex. Moreover, the formation of necrosome can activate phosphoglycerate mutase family member 5 (PGAM5). PGAM5 recruits and activates the mitochondrial fission factor dynamin-related protein 1 (DRP1) by dephosphorylation of serine site [12]. Activation of DRP1 leads to mitochondrial fragmentation and production of reactive oxygen species (ROS), thus resulting in cell necroptosis [13]. In contrast to apoptosis, which is usually immunologically silent, necroptosis usually elicits a pro-inflammatory response. Necroptosis causes rupture of the plasma membrane and release of intracellular components, which contain damage-associated molecular patterns (DAMPs) such as high-mobility group box 1 (HMGB1) protein, eventually leading to a violent inflammatory response.

Emerging evidence has shown that necroptosis has been implicated in neuroinflammation and multiple neurological diseases [14]. Inhibition of necroptosis has been demonstrated to effectively alleviate inflammation and cell damage in the nervous system [15], suggesting that the necroptosis pathway might be an important intervention point for HPA axis activation and inflammation under stress. However, the role of necroptosis in HPA axis activation and inflammation remains obscure. In this study, a piglet model of LPS-induced stress was used to investigate whether necroptosis occurred during HPA axis activation, and the contribution of necroptosis to HPA axis activation and inflammation was further investigated by the prior use of necrostatin-1 (Nec-1), an inhibitor of RIP1.

## 2. Results

### 2.1. LPS Induces Activation of the Hypothalamic-Pituitary-Adrenal Axis

**Figure 1:**
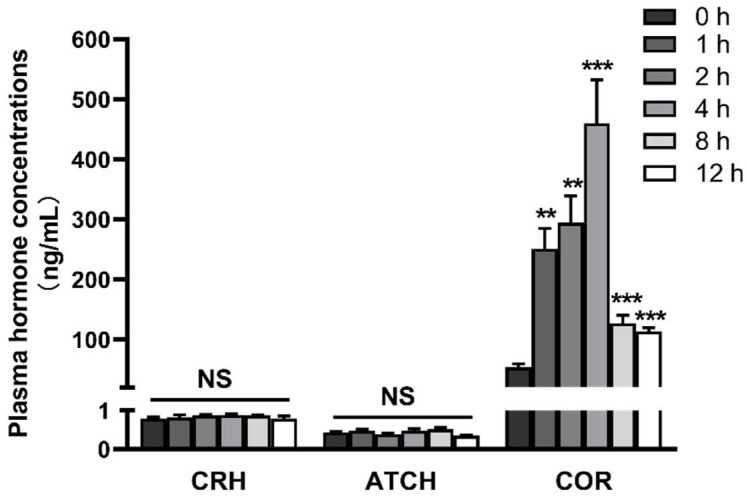
LPS increases the concentrations of cortisol in plasma. The pigs in the control group were sacrificed at 0 h after injection with NaCl solution. The pigs in LPS groups were injected with LPS at 100 μg/kg BW, and then were sacrificed at 1, 2, 4, 8, or 12 h after LPS challenge. Values are means ± SEM, n = 6. ** *p* < 0.01, *** *p* < 0.001, significantly different from the control group (0 h). CRH, corticotropin releasing hormone; ACTH, adrenocorticotrophic hormone.

**Figure 2:**
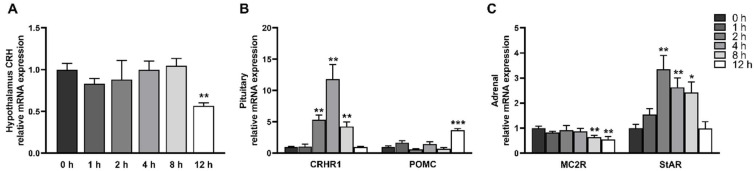
LPS increases the mRNA expression of important components in the hypothalamic-pituitary-adrenal axis: (**A**) *CRH* in the hypothalamus; (**B**) *CRHR1* and *POMC* in the pituitarygland; and (**C**) *MC2R* and *StAR* in the adrenal gland. The pigs in LPS groups were injected with LPS at 100 μg/kg BW, and then were sacrificed at 1, 2, 4, 8, or 12 h after LPS challenge. Values are means ± SEM, n = 6. * *p* < 0.05, ** *p* < 0.01, and *** *p* < 0.001, significantly different from the control group (0 h). *CRH*, corticotropin releasing hormone; *CRHR1*, CRH receptor 1; *POMC*, proopiomelanocortin; *MC2R*, type 2 melanocortin receptor; *StAR*, steroidogenic acute regulatory protein.

Fever, anorexia, shivering, and vomiting were observed in all pigs within 1 h after LPS injection. To investigate the dynamic activation of HPA axis induced by LPS, we measured plasma hormone concentrations including CRH, ACTH, and cortisol, several typical blood biomarkers for activation of HPA axis (Figure 1). There were no significant changes in plasma CRH and ACTH, but cortisol was elevated significantly at 1–12 h after LPS injection (*p* < 0.05) and gradually decreased after reaching the peak at 4 h. In addition, we also measured the mRNA abundance of hypothalamic *CRH*, pituitary CRH receptor 1 (*CRHR1*) and proopiomelanocortin (*POMC*, ACTH precursor peptide), and adrenal type 2 melanocortin receptor (*MC2R*, ACTH receptor) and steroidogenic acute regulatory protein (*StAR*, rate-limiting enzyme involved in steroidogenesis) after LPS injection (Figure 2). No significant change in *CRH* mRNA expression in the hypothalamus was observed between 0–8 h, but the mRNA expression was reduced at 12 h (*p* < 0.05). In the pituitary gland, *CRHR1* was increased at 2–8 h and reached the peak at 4 h, while *POMC* was significantly higher than the control group at 12 h (*p* < 0.05). The mRNA level of *StAR* in the adrenal glands was also increased first and then decreased to the normal level, presenting a significant increase at 2–8 h. MC2R mRNA expression was reduced at 8–12 h (*p* < 0.05).

### 2.2. LPS Induces Dynamic Inflammation of Hypothalamus, Pituitary Gland and Adrenal Gland

**Figure 3:**
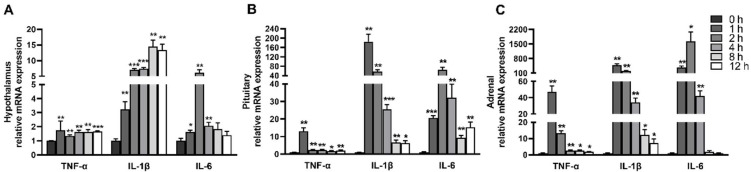
LPS induces dynamic inflammation of the hypothalamus, pituitary, and adrenal gland. (**A**) mRNA expression of *TNF-α*, *IL-1β*, and *IL-6* in the hypothalamus. (**B**) mRNA expression of *TNF-α*, *IL-1β*, and *IL-6* in the pituitarygland. (**C**) mRNA expression of *TNF-α*, *IL-1β*, and *IL-6* in the adrenal gland. The pigs in LPS groups were injected with LPS at 100 μg/kg BW, and then were sacrificed at 1, 2, 4, 8, or 12 h after LPS challenge. Values are means ± SEM, n = 6. * *p* < 0.05, ** *p* < 0.01, and *** *p* < 0.001, significantly different from the control group (0 h). *TNF-α*, tumor necrosis factor α; *IL-1β*, interleukin-1β; *IL-6*, interleukin-6.

Stress responses are often accompanied by inflammatory responses. To investigate the dynamic effect of LPS on the inflammatory response of the hypothalamus, pituitary gland, and adrenal gland, we measured the mRNA expression of *TNF-α*, *IL-1β,* and *IL-6* (Figure 3). LPS rapidly elevated the proinflammatory cytokines in the hypothalamus, pituitary gland and adrenal gland, and the mRNA levels of *TNF-α*, *IL-1β*, and *IL-6* in these tissues were first increased and then declined. The mRNA level of *TNF-α* in the hypothalamus, pituitary gland, and adrenal gland reached the peak at 1 h after LPS injection (1.74 fold, 13.09 fold and 47.38 fold, respectively). The mRNA level of *IL-1β* in the hypothalamus peaked at 8 h (14.5 fold), and that in the pituitary gland and adrenal gland peaked at 1 h (185.02 fold and 486.34 fold, respectively). The mRNA level of *IL-6* in the hypothalamus, pituitary gland and adrenal gland peaked 2 h after LPS injection (6.15 fold, 64.19 fold and 1635.65 fold, respectively).

### 2.3. LPS Induces Dynamic Necroptosis of Hypothalamus, Pituitary Gland and Adrenal Gland

**Figure 4:**
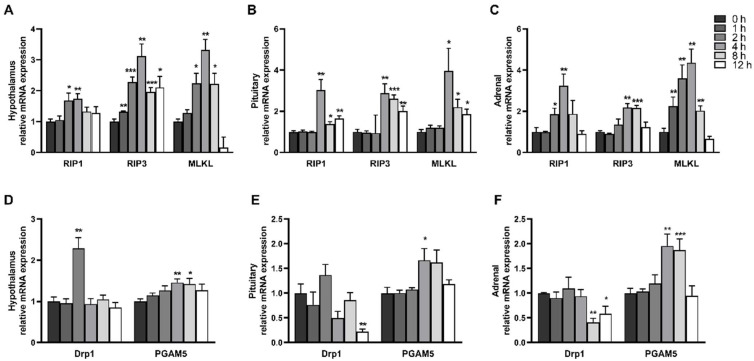
LPS induces dynamic necroptosis of the hypothalamus, pituitary and adrenal gland: (**A**) mRNA expression of *RIP1*, *RIP3*, and *MLKL* in the hypothalamus; (**B**) mRNA expression of *RIP1*, *RIP3*, and *MLKL* in the pituitary gland; (**C**) mRNA expression of *RIP1*, *RIP3*, and *MLKL* in the adrenal gland; (**D**) mRNA expression of *DRP1* and *PGAM5* in the hypothalamus; (**E**) mRNA expression of *DRP1* and *PGAM5* in the pituitary gland; and (**F**) mRNA expression of *DRP1* and *PGAM5* in the adrenal gland. The pigs in LPS groups were injected with LPS at 100 μg/kg BW, and then were sacrificed at 1, 2, 4, 8, or 12 h after LPS challenge. Values are means ± SEM, n = 6. * *p* < 0.05, ** *p* < 0.01, and *** *p* < 0.001, significantly different from the control group (0 h). *RIP1*, receptor interacting protein kinase 1; *RIP3*, receptor interacting protein kinase 3; *MLKL*, mixed lineage kinase domain-like protein; *DRP1*, dynamin-related protein 1; *PGAM5*, phosphoglycerate mutase family member 5.

Necroptosis has been identified in a variety of tissue injuries. To investigate whether LPS elicited necroptosis in the hypothalamus, pituitary gland, and adrenal gland, we measured mRNA levels of *RIP1*, *RIP3*, and *MLKL*, the key components to drive necroptosis (Figure 4A–C). The results showed that LPS increased mRNA levels of *RIP1*, *RIP3*, and *MLKL* in the hypothalamus, pituitary gland and adrenal gland in a time-dependent manner and decreased after reaching the peak. In the hypothalamus, mRNA level of *RIP1* was significantly increased at 2–4 h and peaked at 4 h (1.73 fold), mRNA level of *RIP3* was significantly increased at 1–12 h and peaked at 4 h (3.12 fold), mRNA level of *MLKL* was significantly increased at 2–8 h and peaked at 4 h (3.33 fold) (*p* < 0.05). The mRNA expression of *RIP1*, *RIP3* and *MLKL* in the pituitary gland was significantly increased at 4–12 h (*p* < 0.05) and peaked at 4 h (3.02 fold, 2.88 fold and 3.96 fold, respectively). In the adrenal gland, LPS increased the mRNA expression of *RIP1* between 2–4 h, *RIP3* between 4–8 h, and *MLKL* between 2–8 h, and all three reached the maximum at 4 h (3.25 fold, 2.18 fold, and 4.36 fold, respectively).

We also measured mRNA expression of *DRP1* and *PGAM5*, important components to execute necroptosis (Figure 4D–F). The mRNA expression of *DRP1* in the hypothalamus peaked at 4 h after LPS injection (*p* < 0.05), but no significant increase was observed in the pituitary gland and adrenal gland compared with the control group. In particular, the mRNA level of *DRP1* in the pituitary gland at 12 h and that in the adrenal gland at 8–12 h were significantly decreased (*p* < 0.05). LPS significantly increased *PGAM5* mRNA expression level in the hypothalamus and pituitary gland at 4 h, and in the adrenal gland at 4–8 h (*p* < 0.05).

### 2.4. Nec-1 Inhibits Cell Necroptosis of Hypothalamus, Pituitary Gland and Adrenal Gland

**Figure 5:**
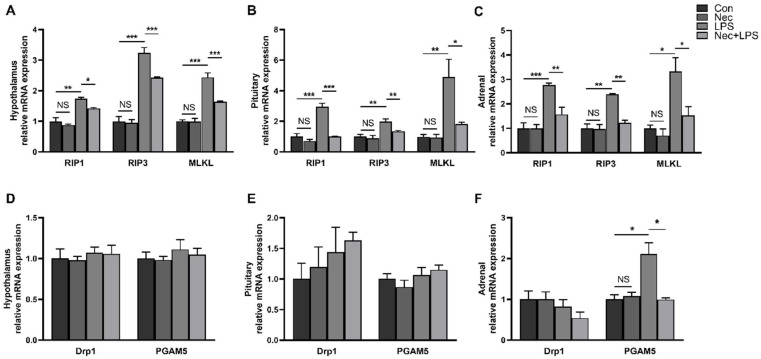
Nec-1 inhibits LPS-induced necroptosis of the hypothalamus, pituitary and adrenal gland. (**A**) mRNA expression of *RIP1*, *RIP3*, and *MLKL* in the hypothalamus. (**B**) mRNA expression of *RIP1*, *RIP3*, and *MLKL* in the pituitary gland. (**C**) mRNA expression of *RIP1*, *RIP3*, and *MLKL* in the adrenal gland. (**D**) mRNA expression of *DRP1* and *PGAM5* in the hypothalamus. (**E**) mRNA expression of *DRP1* and *PGAM5* in pituitary. (**F**) mRNA expression of *DRP1* and *PGAM5* in the adrenal gland. The pigs were pretreated intraperitoneally with Nec-1 at 1.0 mg/kg BW or equal volume of 2% DMSO solution 30 min before the intraperitoneal injection of LPS or saline, and the pigs were sacrificed at 4 h after LPS or saline injection. Values are means ± SEM, n = 7. NS means no significance, * *p* < 0.05, ** *p* < 0.01, and *** *p* < 0.001.

Nec-1 is a small molecule inhibitor that blocks activation of *RIP1* and prevents formation of RIP1/RIP3 complex [16]. Nec-1 has been widely used to inhibits necroptosis of various cells and animal models [15,17,18]. To investigate the role of necroptosis in HPA axis activation, we injected Nec-1 before LPS injection, and measured the mRNA levels of key genes related to necroptosis in the hypothalamus, pituitary gland, and adrenal gland at 4 h after LPS injection (Figure 5). The results showed that LPS significantly increased the mRNA levels of *RIP1*, *RIP3*, *MLKL* and *PGAM5* in the hypothalamus, pituitary gland, and adrenal gland (*p* < 0.05), and the pretreatment of Nec-1 significantly alleviated the effects induced by LPS (*p* < 0.05).

### 2.5. Nec-1 Alleviates LPS-Induced Inflammation of Pituitary Gland and Adrenal Gland

**Figure 6:**
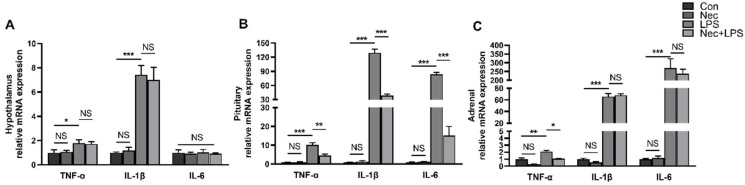
Nec-1 alleviates LPS-induced inflammation of pituitary and adrenal gland: (**A**) mRNA expression of *TNF-α*, *IL-1β*, and *IL-6* in the hypothalamus; (**B**) mRNA expression of *TNF-α*, *IL-1β*, and *IL-6* in the pituitary gland; and (**C**) mRNA expression of *TNF-α*, *IL-1β*, and *IL-6* in the adrenal gland. The pigs were pretreated intraperitoneally with Nec-1 at 1.0 mg/kg BW or equal volume of 2% DMSO solution 30 min before the intraperitoneal injection of LPS or saline, and the pigs were sacrificed at 4 h after LPS or saline injection. Values are means ± SEM, n = 7. NS means no significance, * *p* < 0.05, ** *p* < 0.01, and *** *p* < 0.001.

We measured the mRNA expression of *TNF-α*, *IL-β* and *IL-6* in the hypothalamus, pituitary gland and adrenal gland after pretreatment with Nec-1. In the hypothalamus, the mRNA expression of *TNF-α* and *IL-β* was significantly increased by LPS (*p* < 0.05) but were not alleviated by pretreatment with Nec-1 (Figure 6A). The mRNA expression of *TNF-α*, *IL-β,* and *IL-6* in the pituitary gland and *TNF-α* in the adrenal gland was significantly increased by LPS (*p* < 0.05), while the pituitary gland showed lower mRNA levels of *TNF-α*, *IL-β,* and *IL-6* and the adrenal gland showed lower mRNA level of *TNF-α* when necroptosis was inhibited by Nec-1 (Figure 6B,C).

### 2.6. Inhibition of Necroptosis by Nec-1 Effectively Inhibits LPS-Induced Activation of HPA Axis

**Figure 7:**
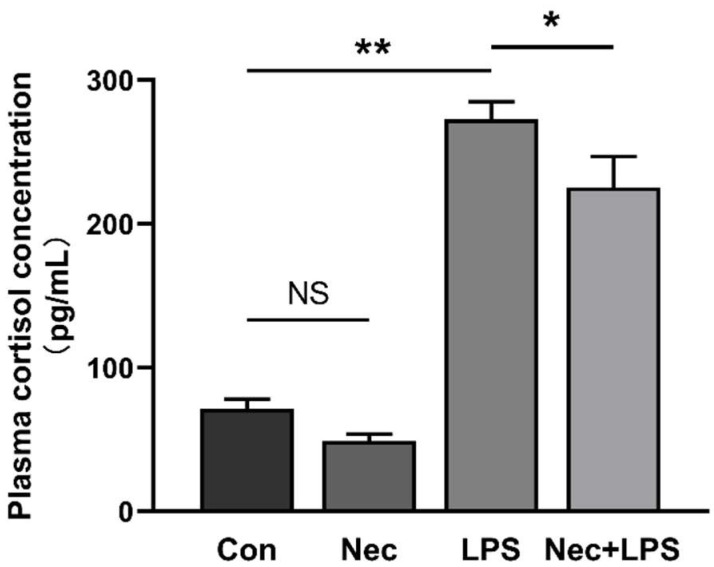
Nec-1 reduces plasma cortisol concentration. The pigs were pretreated intraperitoneally with Nec-1 at 1.0 mg/kg BW or equal volume of 2% DMSO solution 30 min before the intraperitoneal injection of LPS or saline, and the pigs were sacrificed at 4 h after LPS or saline injection. Values are means ± SEM, n = 7. NS means no significance, * *p* < 0.05, and ** *p* < 0.01.

**Figure 8:**
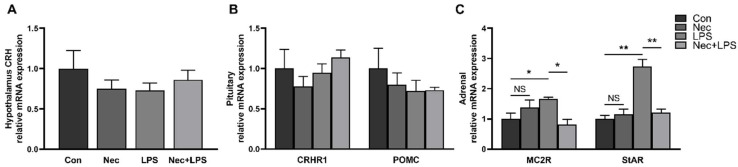
Nec-1 effectively inhibits LPS-induced activation of HPA axis: (**A**) mRNA expression of *CRH* in the hypothalamus; (**B**) mRNA expression of *CRHR1* and *POMC* in the pituitary gland; and (**C**) mRNA expression of *MC2R* and *StAR* in the adrenal gland. The pigs were pretreated intraperitoneally with Nec-1 at 1.0 mg/kg BW or equal volume of 2% DMSO solution 30 min before the intraperitoneal injection of LPS or saline, and the pigs were sacrificed at 4 h after LPS or saline injection. Values are means ± SEM, n = 7. NS means no significance, * *p* < 0.05, and ** *p* < 0.01.

To demonstrate that necroptosis contributes to activation of HPA axis, plasma cortisol concentration (Figure 7) and mRNA levels of hypothalamic *CRH*, pituitary *CRHR1* and *POMC*, and adrenal *MC2R* and *StAR* were determined at 4 h after LPS injection with Nec-1 pretreatment (Figure 8). Plasma cortisol concentration was dramatically increased by LPS, which was alleviated by Nec-1 pretreatment. The mRNA expression of *MC2R* and *StAR* in the adrenal gland was also significantly increased (*p* < 0.05), while inhibition of necroptosis by Nec-1 effectively inhibited the increase of mRNA expression of *MC2R* and *StAR* (with similar mRNA level to the control group).

## 3. Discussion

The HPA axis is a complex regulatory system with direct and feedback interaction composed of the hypothalamus, pituitary gland, and adrenal gland. The HPA axis is an integral part of the neuro-endocrine system, involved in controlling responses to stress or infection and serving to restore the homeostasis, and regulating a variety of physical activities, immune system, emotion, and digestion [19,20]. When the body is exposed to stressors, various cytokines (such as TNF-α, IL-1β, and IL-6) secreted by immune cells act as critical molecular signals that trigger the activation of HPA axis [3]. Our previous studies have found that LPS-induced tissue injury is accompanied by an inflammatory response and necroptosis [21,22]. Thus, we hypothesized that necroptosis might also be involved in HPA axis activation. To verify our hypothesis, piglet, an animal model similar to human physiology was used in the current study.

We firstly investigated the dynamic activation of HPA axis after LPS injection. As expected, HPA axis was activated as manifested by significant increase of plasma cortisol concentration at 1–12 h after LPS injection, and elevated expression of pituitary *CRHR1* and adrenal *StAR*. HPA axis was rapidly activated and gradually restored to normal due to negative feedback system. The concentration of cortisol and the mRNA expression of pituitary *CRHR1* and adrenal *StAR* decreased after peak, which was followed by decreased expression of the hypothalamus *CRH* and adrenal *MC2R*. Actually, study as early as 1957 suggested that LPS could induce the release of ACTH [23]. It has also been speculated that LPS directly mediates the activation of HPA axis, but the target site of LPS activating HPA axis remains controversial. Because LPS could still stimulate the secretion of corticosterone after excision of the hypothalamus, but the effect of LPS on HPA axis could not be observed in excision of the pituitary gland and adrenal gland [24]. Our results also confirmed this, as mRNA expression of pituitary *CRHR1* and adrenal *StAR* was observed to be elevated, but hypothalamus CRH was not elevated after LPS stimulation.

In response to various stressors, cells in the immune system secrete a flood of pro-inflammatory cytokines. Cytokines are also expressed by cells in the nervous system and the long-term chronic over-production of cytokines can cause irreversible damage to the nervous system [25]. Thus, in this study we investigated the dynamic inflammation of the hypothalamus, pituitary gland and adrenal gland induced by LPS challenge. In our study, LPS induced different levels of inflammation at different time points. The mRNA level of *TNF-α* in the hypothalamus, pituitary gland and adrenal gland changed the most rapidly and peaked at 1 h after LPS injection. The maximal response for hypothalamic *IL-1β* was observed later than the maximum response in the pituitary gland and adrenal gland (8 h in the hypothalamus and 1 h in the pituitary gland and adrenal gland). And the maximal response for IL-6 in the hypothalamus, pituitary gland and adrenal gland appeared at 2 h. The mRNA expression of *TNF-α*, *IL-1β,* and *IL-6* in HPA axis was significantly altered, but interestingly, the inflammatory response in the adrenal glands was more intense than that in the brain. It has been reported that only a very minimal percentage of LPS peripherally injected can cross the blood-brain barrier [26]. It suggests that peripheral administration of LPS mainly cause brain inflammation through indirect manner. The immune system is stimulated by LPS challenge and inflammatory mediators are overproduced, and peripheral cytokines can access to the central nervous system directly [27] or further cause the secretion of cytokines of cells in the central nervous system [28], eventually lead to inflammation of the brain. Thus, the inflammatory response in the abdominal organs may be more intense than brain when LPS is intraperitoneally injected.

It is worth noting that the mRNA expression of *TNF-α*, *IL-1β,* and *IL-6* was elevated very rapidly, with significant changes occurring within 1 h after LPS injection and most of them peaking within 1–2 h, whereas the mRNA expression of important molecules in the HPA axis was not increased significantly until 2 h after LPS injection. These results indicated that the secretion of cytokines was earlier than the activation of HPA axis, suggesting that the secretion of cytokines under LPS challenge activated the HPA axis. In fact, this hypothesis was put forward initially by Rivier et al. in 1989 [29] and has been widely demonstrated by many studies [30,31].

Our results suggest that LPS-induced inflammation and HPA activation were accompanied by necroptosis. Moreover, mRNA expression of *RIP1* in the hypothalamus and *MLKL* in the adrenal gland were significantly increased at 1–8 h, indicating that necroptosis occurred rapidly in acute tissue damage. The dynamic change trend of necroptosis was the same as that of inflammation level and HPA axis activation, which firstly increased and then decreased. The occurrence of necroptosis in nervous system damage has been confirmed in several studies [32,33,34]. RIP1 was activated in human AD pathological samples [10]. RIP3 has been confirmed contributing to brain damage after ischemia-reperfusion (IR) by inducing necroptosis [35]. In addition, recent studies have shown that human AD patients with higher protein expression of necroptotic proteins RIP1 and MLKL had lower brain weight, suggesting increased neuronal death [36]. Furthermore, the inhibition of RIP1 reduced neuronal death by approximately 10% in murine models of AD [37]. These studies and our current study suggest that necroptosis is involved in the aggravation of inflammation and progression of tissue damage in the brain.

In order to further explore the role of necroptosis in HPA axis activation, we used Nec-1 (an inhibitor of RIP1) before LPS injection. Interestingly, when necroptosis is suppressed by Nec-1, inflammation of pituitary gland and adrenal gland was effectively alleviated, as evidenced by lower expression levels of *TNF-α*, *IL-β* and *IL-6* in the pituitary gland and *TNF-α* in the adrenal gland in piglets treated with Nec-1 than control group. In addition, Nec-1 also mitigated activation of the HPA axis, as evidenced by lower plasma cortisol concentration and lower mRNA expression of adrenal *MC2R* and *StAR* in Nec-1 treated piglets. Nec-1 has been widely used to investigate the role of RIP1 and necroptosis in physiology and disease due to its effective inhibition of RIP1 in both murine and human [38]. Ito et al. blocked oligodendrocyte death, microglial inflammation, and axonal degeneration in *Optn^−/−^* mice with Nec-1, while the same effect was also observed by RIP3 deficiency [14]. Nec-1 also ameliorated neurovascular injury in intracerebral hemorrhage mice induced by collagenase [39] and inflammatory response and improved cognitive function in chronic ischaemic stroke mice [15]. In addition to inhibitors targeting RIP1 that have a relieving effect on inflammation and injury in the nervous system, inhibitors, or kinase-dead mutations in RIP1, RIP3 and MLKL can also be effective [32,40,41].

A number of studies gave evidence that early functional alterations usually precede neuronal cell death [42,43]. However, various approaches that inhibit cell death can ameliorate the development of neurological diseases [41,44]. RIP1 kinase-dependent necroptosis is a major manner of cell death in the nervous system in response to extracellular inflammatory signals [45]. Moreover, necroptosis has been implicated in a variety of neurodegenerative diseases, including amyotrophic lateral sclerosis (ALS), multiple sclerosis (MS), PD, and AD [14,33,46,47]. In our study, necroptosis was demonstrated to be involved in LPS-induced inflammation and activation of the HPA axis, and the use of Nec-1 could alleviate inflammation and HPA axis activation. It suggested that the necroptosis signaling pathway may be an important point to intervene in the occurrence of inflammation and HPA axis activation.

There are a few limitations in this study. A potential problem is that only one dose of Nec-1 was used in this study, and higher or lower doses of Nec-1 may have different effects. On the other hand, the inhibitory specificity of Nec-1 is limited because Nec-1 blocks both RIP1 and indoleamine 2,3-dioxygenase (IDO) [48]. IDO can also be induced by LPS and accompanied by synthesis and secretion of the proinflammatory cytokines [49]. Thus, the highly specific inhibitors targeting necroptosis such as Nec-1s (an improved analogue of Nec-1 [38], selectively targeting RIP1 but not IDO) should be used in further experiments.

## 4. Materials and Methods

### 4.1. Animals

All experimental procedures were approved by the Animal Care and Use Committee of Wuhan Polytechnic University (Wuhan, China). 28 days old, male, weanling piglets (Duroc × Large White × Landrace) weighing 7.1 ± 0.9 kg were purchased from Aodeng Agriculture and Animal Husbandry Technology Co., Ltd. (Wuhan, Hubei, China). The piglets were individually housed in an environmentally controlled physiology room, and fed a conventional weaned piglet diet for 14 days before experiments, with free access to feed and water. Piglets were food deprived at 8 p.m. the day before experiments began.

### 4.2. Experimental Design

In the first experiment, 36 weanling piglets were randomly divided into six groups including control group and LPS-treated groups slaughtered at five different time points (n = 6). The piglets in LPS-treated groups were injected intraperitoneally (The first pig was injected at 6 a.m.) with *Escherichia coli* LPS (*Escherichia coli* serotype 055: B5, Sigma Chemical Inc., St. Louis, MO, USA) at 100 μg/kg body weight (BW), and then were euthanized at 1, 2, 4, 8, or 12 h after LPS challenge. Based on published studies [50,51] and our preliminary experiments, there was no significant change in inflammation and cortisol of the samples with sham injection at different time points. Thus, the piglets in the control group were euthanized right (0 h) after injection with an equivalent volume of sterile saline. The dose of LPS was administered according to our previous study [52].

In the second experiment, 28 weanling piglets were randomly divided into four groups including control group, Nec-1 group, LPS group, Nec-1+LPS group (n = 7). Nec-1 (MedChem Express, Monmouth Junction, NJ, USA) was pretreated intraperitoneally (The first pig was injected at 6 a.m.) with 1.0 mg/kg BW or an equivalent volume of 2% DMSO solution 30 min prior to intraperitoneal injection of LPS or saline. The piglets were euthanized at 4 h after LPS or saline injection.

### 4.3. Blood and Tissue Sample Collection

Prior to animal sacrifice, blood samples were collected into 10-mL vacuum tubes, and centrifuged to collect plasma (3500 g, 10 min). Plasma was stored at −80 °C for subsequent assay. After blood collection, piglets were euthanized with an intramuscular injection of sodium pentobarbital (80 mg/kg BW). The hypothalamus, pituitary gland, and adrenal gland were collected, frozen immediately in liquid nitrogen, and then stored at −80 °C for further analysis.

### 4.4. Measurement of Plasma Hormone Concentrations

Plasma corticotropin-releasing hormone (CRH), adrenocorticotrophin (ACTH), and cortisol concentrations were determined by commercially available ^125^I RIA kits (Beijing North Institute of Biotechnology Co., Ltd., Beijing, China) according to the manufacturer’s instructions.

### 4.5. Quantitative Real-Time PCR

Total RNA isolation, quantification, reverse transcription, and real-time PCR were performed as the methods described by Liu et al. [50]. The specific primer sequences used are shown in Table 1. The expression of the target genes relative to a reference gene (*GAPDH* or *β-actin*) was analyzed by the 2^−^^△△CT^ method.

### 4.6. Statistical Analysis

All data were presented as means ± standard errors (SEM). In the first experiment, the data were evaluated using independent-sample Student s t test. In the second experiment, the data were evaluated by ANOVA using the general linear model procedures of Statistical Package for Social Sciences (SPSS Version 18). Post hoc testing was conducted using Duncan’s multiple comparison tests. *p* ≤ 0.05 was considered to be statistically significant.

## 5. Conclusions

In summary, our results demonstrate that necroptosis is activated simultaneously with activation of the HPA axis and neuroinflammation induced by LPS. Nec-1, the inhibitor of necroptosis, can inhibit activation of the HPA axis and alleviate inflammation. Based on these results, we conclude that necroptosis is present and contributes to HPA axis activation and inflammation induced by LPS. This study provides evidence that necroptosis may be an important intervention target for inhibiting HPA axis activation and alleviating injury caused by stressors.

## Figures and Tables

**Table 1:** Primer sequences used in real-time PCR.

Gene	Forward (5′-3′)	Reverse (5′-3′)
*TNF-α*	AAGACACCATGAGCACTGAGA	CGACCAGGAGGAAGGAGAAG
*IL-1β*	GCTAACTACGGTGACAACAATAATG	CTTCTCCACTGCCACGATGA
*IL-6*	AAGGTGATGCCACCTCAGAC	TCTGCCAGTACCTCCTTGCT
*RIP1*	ACATCCTGTACGGCAACTCT	CGGGTCCAGGTGTTTATCC
*RIP3*	CTTGTTGTCTGTCCGTGAGC	GAGGAGGTTGGGCTGTTGA
*MLKL*	TCTCGCTGCTGCTTCA	CTCGCTTGTCTTCCTCTG
*DRP1*	TGTGGGCTGCAGGTCATTA	TTGCGCTGGGACATTTTAGC
*PGAM5*	TCTTCATCTGCCACGCCAAT	GGTGATGCTGCCGTTGTTG
*CRH*	CCGCCAGGAGGCACCCGAGAGG	GCCAAACGCACCGTTTCACTTC
*CRHR1*	CTCATCTCCGCCTTCATCCT	CCAAACCAGCACTTCTCATT
*POMC*	AGTAACTTGCTGGCGTGCAT	GAAGTGGCCCATGACGTACT
*MC2R*	TGTTCCCGCTGATGCTGGTGTT	GGGGTCAGCTGGGCAGAGTGTC
*StAR*	GGAGAGCCGGCAGGAGAATG	CTTCTGCAGGATCTTGATCTTCTTG
*GAPDH*	CGTCCCTGAGACACGATGGT	GCCTTGACTGTGCCGTGGAAT
*β-actin*	TGCGGGACATCAAGGAGAAG	AGTTGAAGGTGGTCTCGTGG

*CRH*: corticotropin-releasing hormone; *CRHR1*: CRH receptor 1; *DRP1*: dynamin-related protein 1; *IL*: interleukin; *MC2R*: type 2 melanocortin receptor; *MLKL*: mixed-lineage kinase domain-like protein; *PGAM5*: phosphoglycerate mutase family member 5; *POMC*: proopiomelanocortin; *RIP*: receptor interacting protein kinase; *StAR*: steroidogenic acute regulatory protein; *TNF-α*: tumor necrosis factor-α.
